# Effects of cell size on incidence of chromosome aneuploidy: Analysis using a cultured cell model

**DOI:** 10.20407/fmj.2026-004

**Published:** 2026-09-25

**Authors:** Yoshimi Kato, Makiko Tsutsumi, Takuya Abe, Kazuo Yamamoto, Junichiro Yoshimoto, Takayuki Kannon, Hiroki Kurahashi

**Affiliations:** 1 Division of Molecular Genetics, Center for Medical Science, Fujita Health University, Toyoake, Aichi, Japan; 2 Office of Research Administration, Fujita Health University, Toyoake, Aichi, Japan; 3 Department of Biochemistry, Tohoku Medical and Pharmaceutical University, Sendai, Miyagi, Japan; 4 Biomedical Research Support Center, Nagasaki University Graduate School of Biomedical Sciences, Nagasaki, Nagasaki, Japan; 5 Department of Biomedical Data Science, School of Medicine, Fujita Health University, Toyoake, Aichi, Japan; 6 Laboratory of Data Management and Analysis, Open Facility Center, Fujita Health University, Toyoake, Aichi, Japan; 7 Department of Obstetrics and Gynecology, Kansai Medical University, Hirakata, Osaka, Japan; 8 Clinical Genetics Center, Kansai Medical University Hospital, Hirakata, Osaka, Japan

**Keywords:** Embryonic aneuploidy, Cell size, Early embryo, Mitosis

## Abstract

**Objectives:**

Chromosomal aneuploidy arises from errors in chromosome segregation during cell division, and embryonic aneuploidy is a major cause of implantation failure and miscarriage, which is primarily attributed to maternal meiotic errors. However, recent data accumulated from preimplantation genetic testing have suggested that chromosome segregation errors may also frequently occur during mitotic divisions in early embryos. One proposed factor for these errors is that the cell size in early embryos is larger than that of somatic cells after cleavage, but the exact mechanisms remain unclear.

**Methods:**

To determine the effect of cell size on the incidence of aneuploidy, we established a cultured cell model in which the cell size was artificially enlarged. This model harbored a chromosome carrying the *GFP* gene, and disappearance of GFP fluorescence served as a quantitative marker for aneuploidy events. By overexpressing genes that increase cell size, we measured the cell size and frequency of aneuploidy using flow cytometry.

**Results:**

We observed a significant increase (*p*=3.67×10^–9^) in the incidence of aneuploidy in large cells, which supports the hypothesis that a large cell size characteristic of early embryos contributes to the occurrence of chromosomal segregation errors.

**Conclusions:**

Our finding may partly explain the high prevalence of aneuploid mosaicism in human preimplantation embryos.

## Introduction

Chromosomal aneuploidy arises from errors in chromosome segregation during cell division. Aneuploidy in embryos is a major cause of implantation failure and miscarriage, and many of these cases originate from maternal meiotic errors.^1,2^ In recent years, preimplantation genetic testing (PGT) has been increasingly implemented as part of assisted reproductive technologies. PGT is used to examine numerical or structural chromosomal abnormalities and genetic information at the nucleotide sequence level in embryos obtained through *in vitro* fertilization. To improve pregnancy outcomes, PGT for aneuploidy and structural rearrangements is performed to reduce the risk of miscarriage caused by chromosomal imbalances. This technique involves a comprehensive analysis of the chromosomal copy number using next-generation sequencing of genomic DNA extracted from a few trophectoderm cells of the blastocyst. According to PGT for aneuploidy/structural rearrangement data from approximately 40,000 blastocysts in Japan, 11.7% of embryos showed aneuploid mosaicism.^3^ A review reported mosaic aneuploidy in ≤10% to >20% of embryos.^4^ Mosaic embryos contain a mixture of cells with different chromosomal compositions, suggesting that aneuploidy arises post-fertilization or that partial correction of chromosomal abnormalities may occur during the cleavage divisions of an initially aneuploid zygote. These chromosomal changes predominantly occur during the first three mitotic divisions, which indicates that chromosome segregation errors are frequent even during early mitotic divisions in human embryos.^5,6^

Several factors have been proposed to explain the high incidence of chromosomal segregation errors in early human embryos. One factor is the delayed activation of the embryonic genome until the four- to eight-cell stage, which may result in insufficient maternal mRNA and protein reserves, thereby impairing the proper regulation of cell division.^7,8^ Other contributing factors include the rapid cell cycle and replication stress, which may lead to incomplete DNA replication and subsequent chromosome breakage during cell division.^9^ Another hypothesis suggests that the unusually larger cell size in early embryos than in somatic cells post-cleavage plays a role. Studies on *Caenorhabditis elegans* have investigated the spindle assembly checkpoint (SAC), which is a surveillance mechanism that monitors kinetochore–microtubule attachment and halts mitotic progression until all kinetochores are properly attached to spindle microtubules.^10^ SAC activity was found to increase with successive cell divisions, and checkpoint strength was weaker in larger cells compared with smaller cells.^11^ However, *C. elegans* chromosomes are holocentric, and SAC activity in early mammalian embryos is still unclear.^12^ Notably, a larger cell size was associated with weaker SAC activity in mouse oocytes.^13^ In contrast, in mouse early embryos, a reduction in cell volume at the two-cell stage did not alter SAC failure.^14^ Moreover, early embryos possess unique translational profiles and cell cycle dynamics, causing difficulty in determining the direct effect of cell size on chromosome segregation.

To investigate the relationship between cell size and the propensity for chromosome segregation errors, we developed a cultured cell model in which the cell size was artificially enlarged. Using a fluorescence-based system that enables the simple detection of aneuploidy, we quantified the incidence of chromosomal missegregation. This system used the loss of a GFP (Green Fluorescent Protein) fluorescence signal as an indicator of chromosomal aneuploidy. This study showed that the frequency of aneuploidy increased as the cell size became larger, which provides a basis for discussing how cell size during mitosis may affect the fidelity of chromosome segregation mechanisms.

## Materials and methods

### Cell culture

DT40-T315 cells, which are referred to as DT40 Chr2 (1, 1, mini-GFP),^15^ were cultured in D-MEM/F-12 medium (FUJIFILM Wako Pure Chemical Corporation, Osaka, Japan) at 39.5°C in a humidified atmosphere containing 5% CO_2_. The culture medium was supplemented with 10% fetal bovine serum (Sigma-Aldrich, Tokyo, Japan), 2% chicken serum (Gibco, USA), penicillin/streptomycin mix, 2 mM L-glutamine (Nacalai Tesque, Kyoto, Japan), and 10 μM 2-mercaptoethanol (Abcam, Cambridge, UK). On the basis of the fact that the mini-chromosome carries a puromycin resistance gene, 0.5 μg/ml puromycin (FUJIFILM Wako Pure Chemical Corporation) was added 24 hours before electroporation to select only cells containing the mini-chromosome.

### Plasmid construction and transfection

Plasmids were constructed by inserting cDNA encoding the selected genes^16^ into the ptdTomato-C1 vector (Clontech, CA, USA). The restriction enzyme sites used were *Bgl* II/*Kpn* I for *mPRR16* (encoding Largen) and *mDSC54C* (encoding LargenΔN), *VPS26C*, and *Sal* I/*Apa* I for *FYB1*. The *mPRR16* and *mDSC54C* genes originate from mice, whereas the *VPS26C* and *FYB1* genes originate from humans. Electroporation was performed using the Neon Transfection System (Invitrogen, CA, USA) with parameters set to 1,400 V, 20 ms, and six pulses, as indicated in the manufacturer’s instructions. After washing out puromycin from DT40-T315 cells, 1 μg of plasmid DNA was transfected into 2×10^5^ DT40-T315 cells. The transfected cells were cultured in 24-well plates using medium without antibiotics.

### Flow cytometric analysis

The cells were harvested 24 hours after electroporation, washed twice with 1× PBS (FUJIFILM Wako Pure Chemical Corporation), and resuspended in 1× PBS containing 1% BSA (Sigma-Aldrich) and 0.1 μg/ml DAPI (Sigma-Aldrich). After being incubated at room temperature for 5 minutes, the cell suspension was filtered through a 35-μm cell strainer. Samples were analyzed using the CytoFLEX S multicolor flow cytometer (Beckman Coulter, CA, USA), and data were processed with CytExpert software (Beckman Coulter). Debris was excluded using FSC-A and SSC-A parameters, doublets were removed using FSC-A and FSC-H, and dead cells were gated out using FSC-A and PB450-A on the basis of DAPI staining. GFP-positive cells were detected using the FITC channel and tdTomato-positive cells using the PE channel. FSC-A was used as a relative proxy for cell size to compare cells analyzed under identical flow cytometry conditions. However, notably, FSC-A is not a direct measurement of cell size and can be affected by factors, such as cell morphology and the cell cycle phase.

### Statistical analysis

To quantify the association between tdTomato fluorescence intensity and the GFP status, a logistic regression analysis was performed at the single-cell level. The GFP status was modeled as a binary outcome (GFP-negative=1, GFP-positive=0). The model included the tdTomato fluorescence intensity level, transfected genes, and their interaction as explanatory variables. “Trial” was included as a covariate to account for potential batch effects. Control cells with negative tdTomato fluorescence intensity were used as the reference category. Data were obtained from three independent experiments.

To assess the relationship between the cell size and GFP status, a correlation analysis was performed using Spearman’s rank correlation coefficient. To model the relationship between FSC-A and the percentage of GFP-negative cells, a nonlinear regression analysis was conducted using the Michaelis–Menten equation: [%GFP-negative cells]=V_max_×[FSC-A]/(K+[FSC-A]).Model parameters (V_max_ and K) were estimated with 95% confidence intervals. Data from three independent experiments were analyzed. To perform comparisons between two groups, statistical significance was assessed using the unpaired two-tailed Student’s *t*-test.

## Results

To enlarge the cell size, we initially attempted to overexpress genes identified from a screen for human “stay-large” cells (ISLC genes), which are associated with increased cell volumes.^16^ The ISLC genes used in the present study included *PRR16*, *FYB1*, and *VPS26C* (Table 1).^16–18^ Alternative splicing of *PRR16* leads to Largen and LargenΔN isoforms. To date, none of these genes have been shown to play a direct role in chromosome segregation or cell cycle regulation. Plasmids were constructed to express the fusion proteins of each gene with tdTomato under the control of the CMV promoter, and these plasmids were introduced into DT40-T315 cells.

DT40-T315 cells are derived from chicken B cells and possess a trisomy of chromosome 2, in which one of the three copies has been genetically edited to form a mini-chromosome carrying the *GFP* gene (Figure 1a). The loss of this mini-chromosome results in the absence of GFP fluorescence, which serves as an indicator of aneuploidy.^15^ Plasmids were introduced into DT40-T315 cells via electroporation, and cells were analyzed by flow cytometry 24 hours post-transfection (Figure 1b). tdTomato fluorescence was used as a marker for gene expression, while GFP fluorescence served as a marker for aneuploidy. The cell size was assessed using the FSC-A parameter (Figure 2). The GFP-negative rate was calculated as the percentage of GFP-negative cells among the total number of GFP-positive and GFP-negative cells.

To determine whether the introduction of plasmids expressing ISLC genes increased the size of DT40-T315 cells, we analyzed the relationship between tdTomato fluorescence intensity and cell size, as indicated by FSC-A values. We categorized tdTomato fluorescence into three levels: negative, weak positive, and positive (Figure 1b). The relationship between fluorescence intensity and FSC-A was then examined (Figure 2a). We found that higher tdTomato fluorescence intensity was associated with higher FSC-A values.

Cells introduced with ISLC genes and showing positive tdTomato fluorescence showed higher mean FSC-A values than control cells transfected with an empty vector. In comparisons of the percentage of cells with FSC-A values exceeding 5×10^5^, 26% were control cells, whereas cells expressing Largen, LargenΔN, FYB1, and VPS26C accounted for 31%, 28%, 29%, and 33%, respectively. These results confirmed that the overexpression of ISLC genes increased the size of DT40-T315 cells. Notably, these genes are conserved in chickens, suggesting that similar pathways regulating cell size are active in human and avian cells. Although an increase in FSC-A was also observed in control cells with high tdTomato fluorescence, FSC-A values were higher than those in cells subjected to electroporation without introduction of a plasmid (Figure 2b). This finding indicated that plasmid transfection slightly increased the size of DT40-T315 cells.

Having successfully enlarged the cell size, we next examined whether the frequency of aneuploidy was increased in these cells by analyzing the GFP-negative rate in cells expressing tdTomato-fused ISLC proteins. The proportion of GFP-negative cells tended to increase in all samples at higher tdTomato fluorescence intensities (Figure 3), reaching up to a 1.9-fold increase (Supplementary Figure S1). GFP-negative rates tended to be higher in weak positive and positive tdTomato populations in cells expressing tdTomato-fused ISLC proteins than in control cells. Cells expressing Largen showed the highest increase, in which the proportion of GFP-negative cells was approximately1.4-fold higher than that of control cells in the tdTomato-positive population (Figure 3). Although comparisons based on population means showed limited statistical significance, a logistic regression analysis at the single-cell level showed that the GFP-negative status was significantly associated with tdTomato fluorescence intensity and ISLC gene transfection (Table 2). Cells with positive tdTomato fluorescence intensity showed significantly increased odds of GFP negativity compared with the reference group (control cells with negative tdTomato fluorescence intensity) (odds ratio, 1.479–2.132; *p*<0.001). Additionally, cells transfected with ISLC genes showed higher odds of GFP negativity relative to control cells. Consistent results were obtained across three independent experiments. Taken together, these results suggest that artificial enlargement of cell size through overexpression of multiple distinct ISLC genes is associated with an increased frequency of chromosomal aneuploidy. Although control cells also showed an increased cell size with higher tdTomato fluorescence (Figure 2a), an accompanying increase in the GFP-negative rate was observed (Figure 3 and Supplementary Figure S1). This finding suggests an association between increased tdTomato fluorescence, cell size, and the likelihood of aneuploidy as reflected by GFP loss.

To further clarify the relationship between cell size and the frequency of aneuploidy, we performed an additional analysis comparing FSC-A values (as a proxy for cell size) and GFP-negative rates, independent of tdTomato fluorescence intensity. Cell size, as measured by FSC-A, was positively correlated with the frequency of GFP-negative cells (Spearman’s *r*_s_=0.58, *p*=3.67×10^–9^; Figure 4). As FSC-A increased, the percentage of GFP-negative cells showed a saturating increase. Nonlinear regression using the Michaelis–Menten model showed an estimated V_max_ of 3.29 (95% confidence interval: 2.43–4.16) and a K value of 4.29×10^5^ (95% confidence interval: 1.78×10^5^–6.80×10^5^). Consistent results were obtained across three independent experiments. However, in populations with mean FSC-A values exceeding 6.0×10^5^, the GFP-negative rate tended to decrease as the cell size increased. This reduction in the GFP-negative rate among excessively large cells suggests that cells exceeding the optimal size for DT40 cease to divide or, if such cells are more susceptible to aneuploidy, are selectively eliminated during proliferation. Collectively, these results suggest an association between cell size and the frequency of aneuploidy, and imply that an increased cell size may contribute to the occurrence of chromosome missegregation.

## Discussion

Using a cultured cell model, this study showed that a large cell size increased the frequency of chromosomal aneuploidy. Methods for enlarging the cell size include cell fusion^13^ and drug-induced senescent cell models.^19^ While these approaches effectively increase the cell size, they may impose considerable stress on cells. In contrast, the overexpression of genes to enlarge the cell size in the present study did not achieve the same degree of enlargement as that in early embryonic cells and it was not as effective as the above-mentioned methods. However, gene overexpression resulted in a moderate increase in cell size that relied on the physiological functions of gene expression. Therefore, the effects of cell size may be investigated under conditions similar to those of normal cells. Although plasmid transfection alone caused a modest increase in FSC-A, the consistently greater increases observed upon overexpression of the genes used in this study indicate effects beyond a general transfection-related phenomenon.

The precise molecular mechanism by which aneuploidy increased with the cell size in DT40 cells in this experimental system remains unclear. Larger cells are likely to reach the size required for division more quickly, resulting in a shortened G1 phase, a faster cell cycle, and consequently, more rapid division.^20^ This rapid division may lead to an increased number of cell divisions and, consequently, more opportunities for aneuploidy to occur. However, in cells overexpressing Largen in a previous study, the proliferation rate was slower than that of control cells, and no significant difference in cell cycle progression was observed.^16^ These findings suggest that increased proliferation is not the cause of aneuploidy in Largen-overexpressing DT40 cells.

Another possible cause of aneuploidy increasing with the cell size is impairment of the SAC. A previous study of tetraploid DLD-1 cells showed that clones with smaller cells took longer to complete mitosis and showed stronger SAC surveillance than larger clones.^21^ Additionally, in mouse oocytes with a 50% smaller cytoplasmic volume, concentrations of active Mad2, a SAC protein, increased, which delayed the onset of anaphase and improved bi-orientation of chromosomes. In contrast, when the volume was doubled, Mad2 was diluted and SAC stringency decreased.^13^ Therefore, an increased cell size may impair the SAC, thereby inducing aneuploidy. Another potential mechanism is the imbalance between the cell size and the metaphase spindle size. If the spindle size is insufficient in enlarged DT40 cells, microtubules may fail to capture chromosomes, leading to chromosome loss, as demonstrated in experiments using HeLa cells.^22^ The structure and size of the spindle also play a crucial role in SAC regulation, and these factors may interact with cell size to affect the strength of the SAC.^21^

The present results support the hypothesis that a large cell size contributes to susceptibility to aneuploidy in early embryos. This aligns with previous findings in early embryonic studies showing a decline in SAC function with increasing cell size in early *C. elegans* embryos^11^ and spindle size having an upper limit that does not correlate with cell size during early cleavage stages in *Xenopus laevis* and mice.^23,24^ SAC functionality in early mammalian embryos remains largely unclear and is considered to be closely related to the occurrence of aneuploidy.^12^ A more detailed understanding of the effect of cell size is essential for determining this relationship.

Notably, DT40 cells differ substantially from human embryos in several cellular properties, including lineage identity and developmental context. Additionally, the mini-chromosome model used in this study represents a simplified and artificial chromosomal system that may not fully recapitulate the structural complexity, chromatin organization, or regulatory dynamics of endogenous chromosomes. Accordingly, caution is warranted when extrapolating these findings directly to human embryonic development, and the present results should be interpreted as mechanistic insights derived from this experimental model. In this study, the loss of GFP signal was used as a surrogate marker for mini-chromosome loss and thus aneuploidy. This readout has been used previously in the mini-chromosome system, including in a previous study by our research group.^15^ However, direct confirmation of the copy number was not performed in the present study. Therefore, alternative explanations, such as transcriptional silencing of GFP or transfection-related cellular stress, cannot be fully excluded. The observed GFP loss should be interpreted as reflecting relative changes in mini-chromosome stability rather than absolute measurements of the chromosome copy number.

The further clarification of mechanisms underlying aneuploidy in early embryos may lead to improvements in culture conditions and the development of safe additives. These outcomes could ultimately reduce chromosome segregation errors in *in vitro* fertilized embryos and increase the number of embryos suitable for transplantation. These advances may reduce the risk of miscarriage and increase pregnancy and birth rates in reproductive medicine.

## Figures and Tables

**Figure 1:**
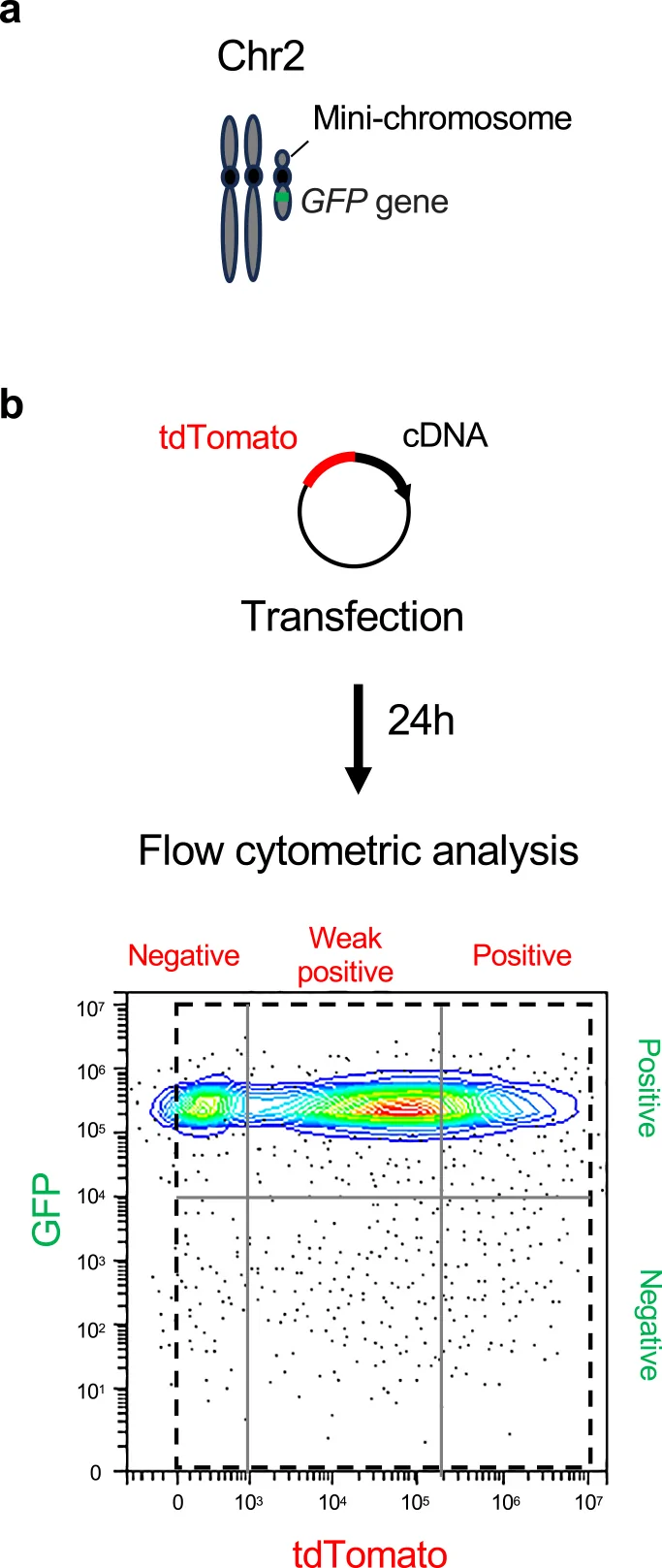
Experimental system for detecting aneuploidy. a) Schematic representation of chromosome 2 in DT40-T315 cells used to detect aneuploidy. The mini-chromosome carries the *GFP* gene, causing the cells to show green fluorescence. b) DT40-T315 cells were transfected with a plasmid containing the ISLC gene and were analyzed by flow cytometry 24 hours later. Cells that received the plasmid expressed tdTomato. Cells that lost the mini-chromosome no longer expressed GFP. The lower panel shows a representative flow cytometric analysis. As indicated by a contour plot, the cell population enclosed by the dashed line was analyzed and classified as GFP-positive or GFP-negative cells.

**Figure 2:**
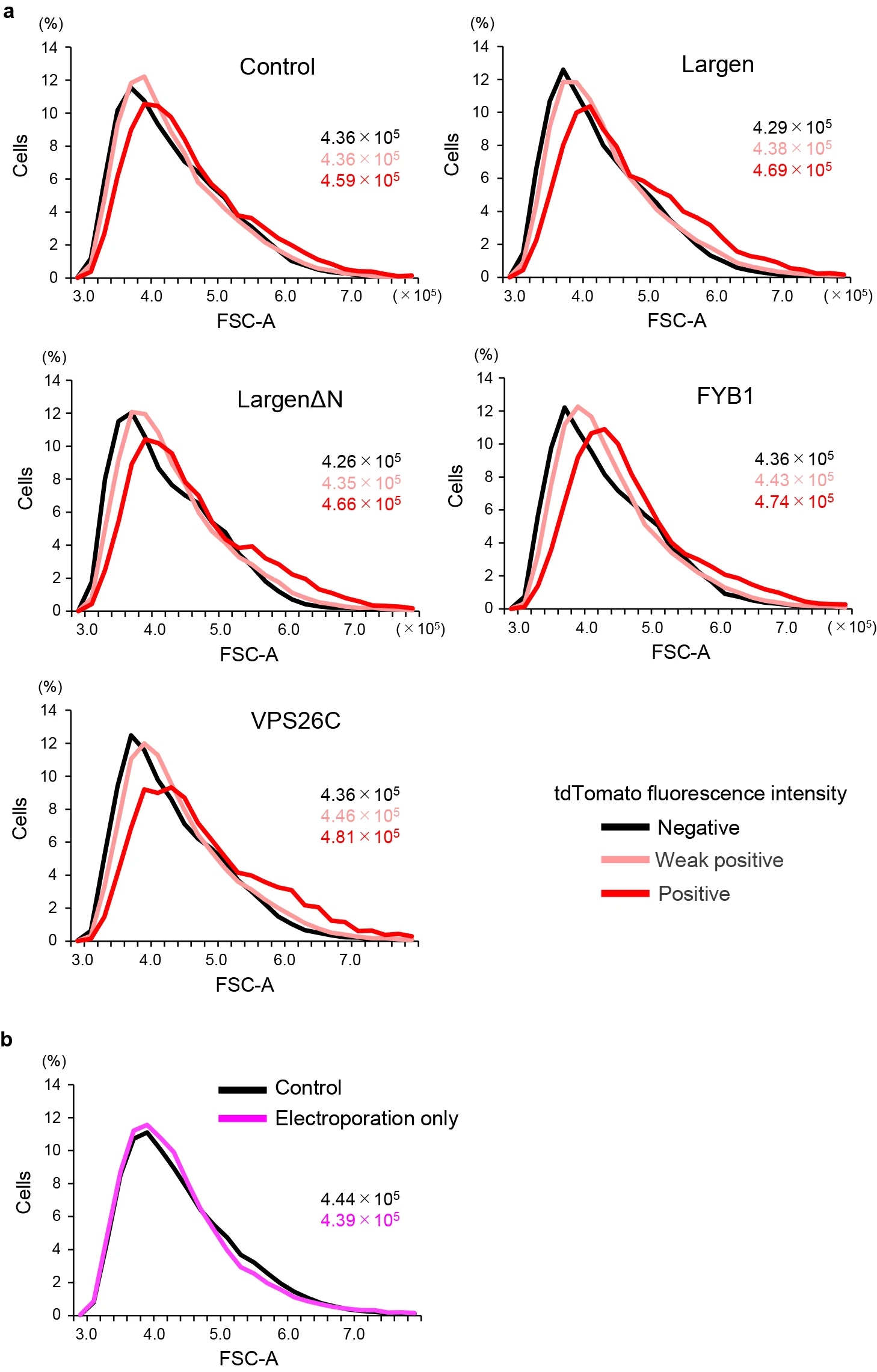
Distribution of cell sizes (FSC-A) according to tdTomato fluorescence intensity. a) Histograms of FSC-A values by tdTomato fluorescence intensity, shown as the percentage of cells. The numbers in the figure indicate mean FSC-A values. Representative data from three independent experiments are shown. b) Histograms of FSC-A values for control cell populations and those subjected only to electroporation, without distinguishing tdTomato fluorescence intensity. The numbers in the figure indicate mean FSC-A values.

**Figure 3:**
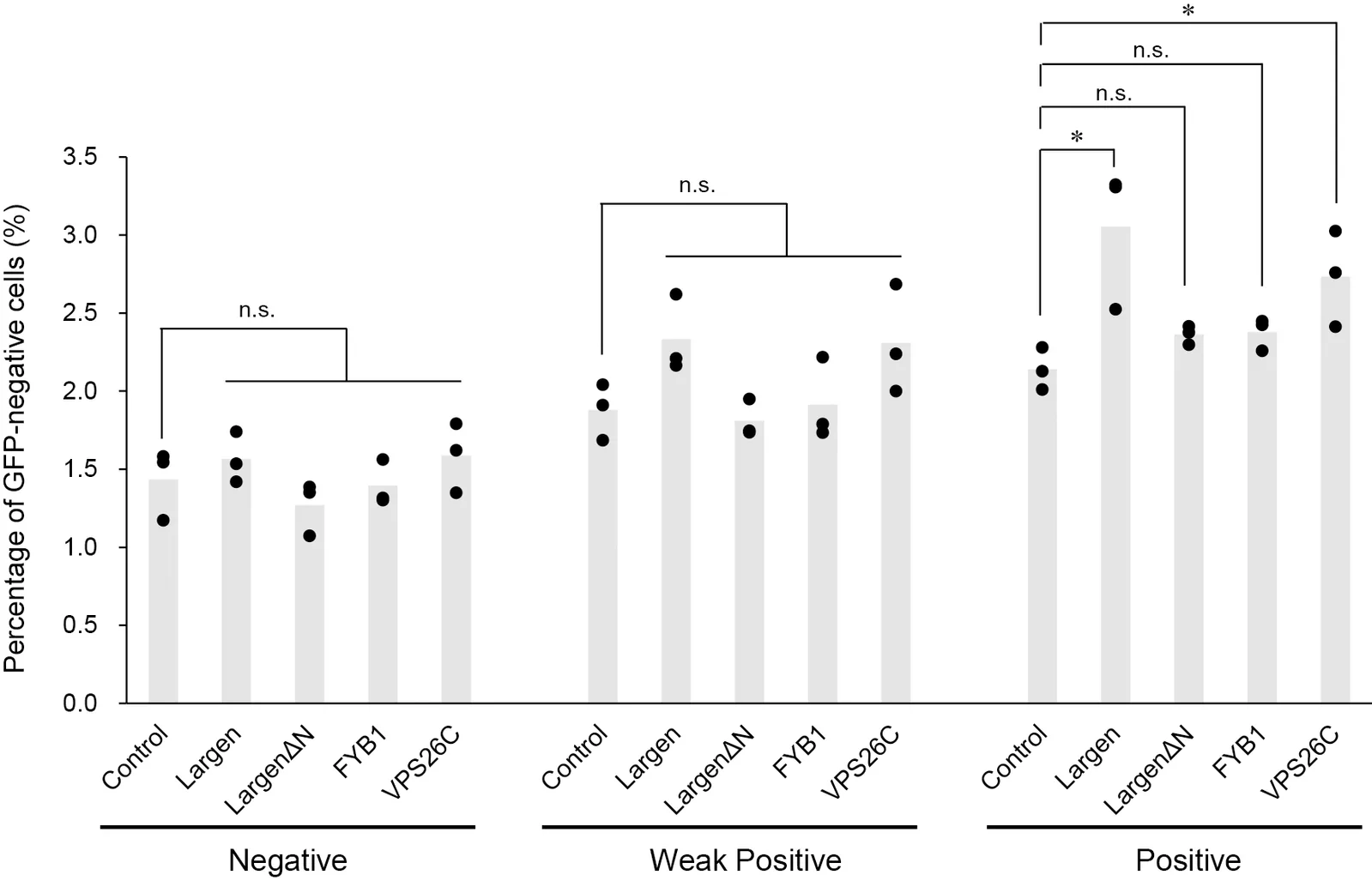
Percentage of GFP-negative cells according to tdTomato fluorescence intensity. Individual dots represent GFP-negative rates in cell populations with negative, weak positive, and positive tdTomato fluorescence. Bars indicate the mean values from three independent experiments. *p<0.05; n.s., not significant (Student’s *t*-test).

**Figure 4:**
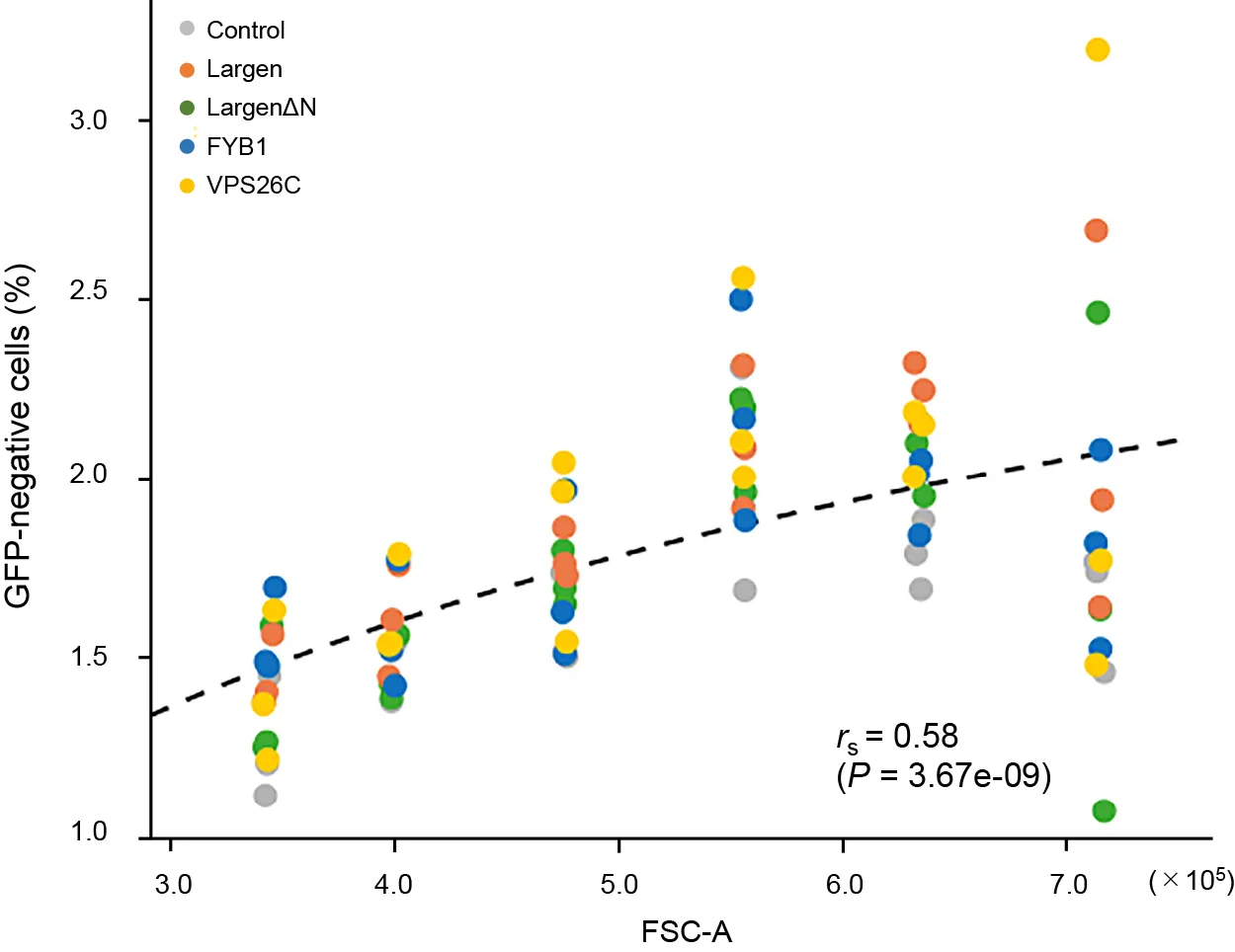
Relationship between cell size (FSC-A) and the frequency of GFP-negative cells. Scatter plots show the percentage of GFP-negative cells as a function of FSC-A. The dashed line represents the fitted curve obtained by nonlinear regression. Data from three independent experiments are shown.

**Table 1:** ISLC genes used in this study.

Gene	Function	Reference
*PRR16 (Proline rich 16)*	Encodes Largen and LargenΔN via alternative splicing. Functions as a regulator of cell size, and affects mitochondrial activity.	^16^
*FYB1 (FYN binding protein-1)*	Related to T cell signaling.	^17^
*VPS26C (VPS26 endosomal protein sorting factor C)*	Component of the retriever complex involved in endosomal cargo retrieval and recycling.	^18^

**Table 2:** Logistic regression analysis of factors associated with the GFP-negative status.

Variable		Odds ratio	95% CI	*p* value
Protein	tdTomato intensity			
Control	Negative	1	Reference	
	Weak positive	1.302	1.246–1.360	<0.0001
	Positive	1.479	1.412–1.548	<0.0001
Largen	Negative	1.097	1.055–1.140	<0.0001
	Weak positive	1.651	1.584–1.722	<0.0001
	Positive	2.132	1.977–2.299	<0.0001
LargenΔN	Negative	0.879	0.837–0.924	<0.0001
	Weak positive	1.249	1.203–1.296	<0.0001
	Positive	1.605	1.528–1.686	<0.0001
FYB1	Negative	0.975	0.920–1.034	0.3986
	Weak positive	1.361	1.303–1.421	<0.0001
	Positive	1.633	1.557–1.713	<0.0001
VPS26C	Negative	1.122	1.076–1.171	<0.0001
	Weak positive	1.649	1.580–1.720	<0.0001
	Positive	1.88	1.719–2.056	<0.0001
Trial	#1	1	Reference	
	#2	1.041	1.009–1.073	0.0106
	#3	1.185	1.156–1.215	<0.0001

CI, confidence interval.

## Data availability

The datasets used and/or analyzed during the current study are available from the corresponding author on reasonable request.
