# Supplementary material for: Effects of cell size on incidence of chromosome aneuploidy: Analysis using a cultured cell model
Source: Fujita Med J. 2026 Sep 25;12(4):297–303. doi: 10.20407/fmj.2026-004 (PMC13724193; doi:10.20407/fmj.2026-004)
Supplement: Supplementary file 1 [file fmj-12-297_s1.pdf]

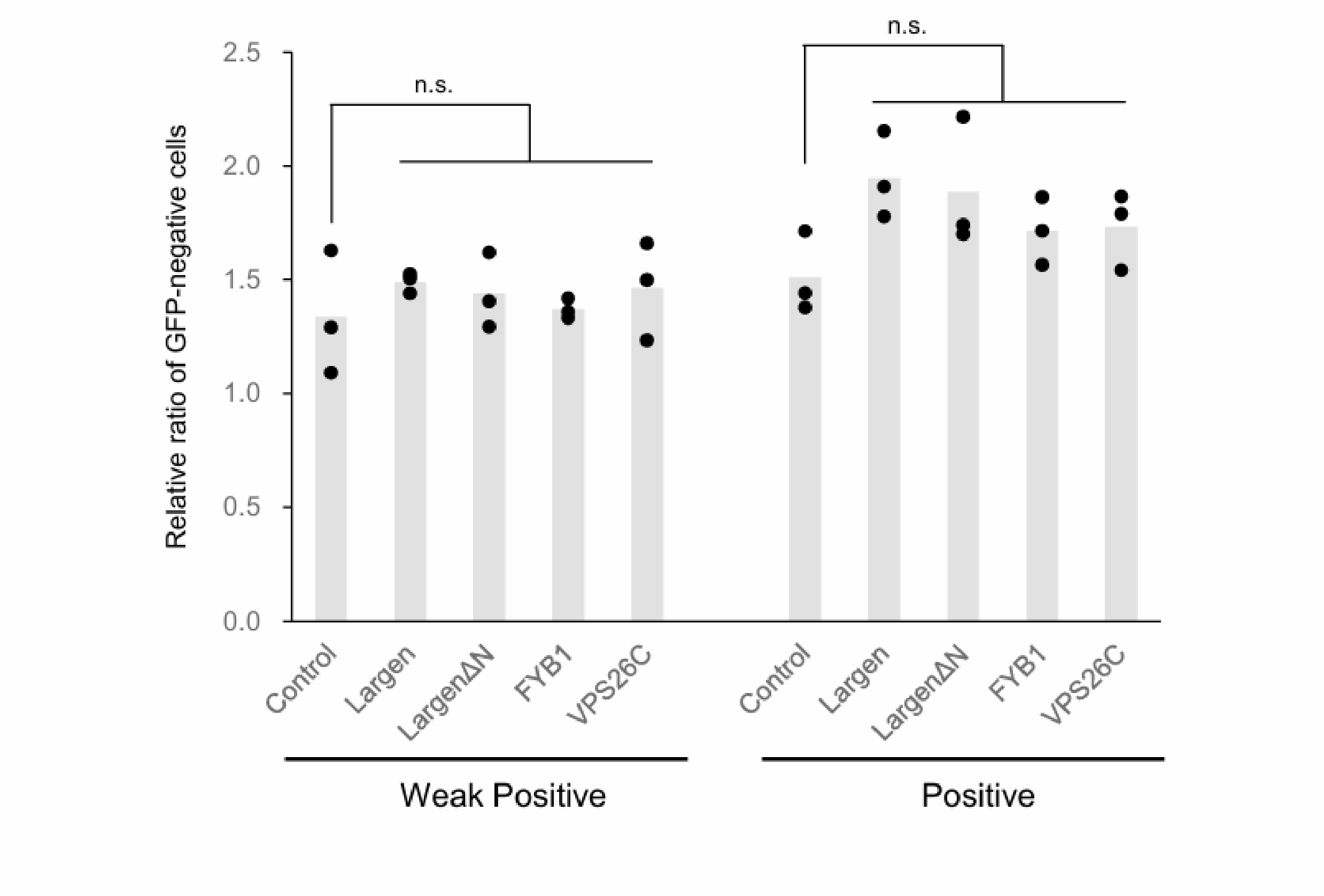

Supplementary Figure S1

Relative ratio of GFP-negative cells according to tdTomato fluorescence intensity.

Individual dots represent the ratios of GFP-negative cells in populations with Weak Positive and Positive tdTomato fluorescence relative to those with Negative fluorescence. Bars indicate the mean values from three independent experiments. n.s., not significant (Student's *t*-test).
